# Nursing-led tele-coaching for enhancing self-efficacy and lifestyle adherence among gestational diabetic women: tele-nursing versus traditional nursing intervention program

**DOI:** 10.1186/s12912-026-04290-y

**Published:** 2026-01-29

**Authors:** Soheir Mahmoud Abd El-Hamid, Faiza Mohamed EL-Said, Hend Reda Ali El-kest, Ayman Muhammad Kamel Senosy, Amina Abdelrazek Aldeeb, Manar Gamal Mohamed

**Affiliations:** 1 Maternal and Neonatal Health Nursing, Faculty of Nursing, Tanta University, Tanta, Egypt; 2 Community Health Nursing, Faculty of Nursing, Tanta University, Tanta, Egypt; 3 Medical Surgical Nursing Department, Faculty of Nursing, Ain Shams University, Cairo, Egypt; 4 Faculty of Nursing, Galala University, Suez, Egypt; 5 Obstetric and Maternity Nursing, Faculty of Nursing, Galala University, Suez, Egypt

**Keywords:** Tele-nursing, GDM, Healthy lifestyle

## Abstract

**Background:**

Increased maternal morbidity and mortality as well as poor neonatal outcomes are two major public health concerns associated with gestational diabetes mellitus (GDM) GDM. Maternal health can be improved using tele-nursing, especially in rural areas where access to healthcare is limited. The study’s aim is to assess the effects of tele-nursing with standard nursing care as a novel strategy for improving the lifestyle, self-efficacy, and satisfaction of women with gestational diabetes.

**Design:**

A quasi-experimental design was used.

**Setting:**

Three major hospitals in Tanta City’s outpatient clinics served as the study’s prenatal care units. Sample: The study included two hundred mothers with gestational diabetes who were split into two groups: Tele-nursing and Traditional.

**Tools:**

Four tools were used for data collection: Tool I: Structured interviewing questionnaire for knowledge assessment, Tool II: Health promotion lifestyle profile scale, Tool III: Self-efficacy of health activities, and Tool IV: Satisfaction of Women the Likert scale.

**Results:**

The study revealed that there was a statistically significant increase in the total knowledge scores for both groups, adherence to routine blood testing, and compliance with healthy lifestyle practices after the intervention; however, the improvement of the tele-nursing group’s improvement was more noticeable. Moreover, analysis revealed a significant positive correlation between the total score of healthy lifestyle practices and the total knowledge score in both groups, indicating that higher knowledge levels were associated with better adherence to healthy lifestyle behaviors. This suggests that enhancing women’s knowledge through targeted interventions such as tele-nursing can play a pivotal role in promoting healthier practices and improving self-care outcomes.

**Conclusion:**

The study findings indicate that tele-nursing support can substantially improve health promotion lifestyle profiles, self-efficacy, and maternal satisfaction, while effectively maintaining optimal blood glucose control in mothers with gestational diabetes. Additionally, it was found to enhance adherence to scheduled antenatal visits, highlighting its value as a supportive intervention in maternal healthcare.

**Recommendations:**

Based on the evidence of tele-nursing effectiveness, health policymakers should integrate tele-nursing into routine care protocols for gestational diabetic women across all healthcare levels. Implementing this approach can enhance patient monitoring, improve treatment adherence, reduce complications, and optimize resource utilization, ultimately contributing to better maternal and neonatal health outcomes.

**Clinical trial number:**

Not applicable.

## Introduction

Gestational diabetes mellitus is a pressing global health concern, owing not only to its increasing occurrence but also to its potential adverse effects on maternal and neonatal health. It’s a type of carbohydrate intolerance that appears or is initially identified during pregnancy, and its high prevalence is caused by several variables, such as a sedentary lifestyle, obesity, repeated gestations, advanced maternal age, polycystic ovarian syndrome, and inadequate sleep Benton et al. [1]. It is estimated that up to 80% of diabetes cases can be prevented through proactive strategies such as improving nutritional awareness, consistent blood glucose monitoring, maintaining optimal glucose control, decreasing dependence on insulin therapy, and moderating carbohydrate consumption Mittal et al. [2]. The effect of educational programs, particularly those that use the Health Promotion Lifestyle Profile II (HPLPII), on encouraging preventive health behaviors has been the subject of much research Zhong et al. [3].

Gestational diabetes is associated with a wide range of maternal and neonatal complications spanning the antepartum, intrapartum, and postpartum periods. These include hypertensive disorders, increased rates of cesarean delivery, labor dystocia, macrosomia, preterm birth, and respiratory distress in newborns. Beyond immediate pregnancy outcomes, it also predisposes children born to affected mothers to long-term health risks, such as sustained insulin resistance, an elevated likelihood of developing type 2 diabetes, obesity, and metabolic syndrome during childhood and adolescence. Such complications highlight the critical need for early detection, effective management, and ongoing follow-up to reduce both short- and long-term adverse effects Karkia et al. [4].

When gestational diabetes mellitus is diagnosed, there is a window of opportunity to improve pregnancy outcomes and encourage dietary and lifestyle changes that will improve general health. Making healthy lifestyle choices including eating a balanced diet, exercising frequently, and controlling their weight can significantly reduce the risk of type 2 diabetes in the future for women with a history of GDM., El Sayed et al. [5].

Lifestyle is a significant determinant in the prevention and management of GDM, with research indicating that more than 70% of diseases are at least partially influenced by lifestyle behaviors. Current strategies for preventing GDM increasingly focus on delivering comprehensive education that promotes healthy living, emphasizing the adoption of regular physical activity and the maintenance of a well-balanced, nutrient-rich diet. By fostering these habits, the risk of developing GDM can be substantially reduced, ultimately improving both maternal and neonatal health outcomes Zakaria et al. [6].

Lifestyle modification is considered the most effective strategy for managing GDM. Central to this approach is a balanced diet, emphasizing healthy nutrition and appropriate weight gain, with careful regulation of carbohydrate intake particularly its quantity, distribution, and form. The inclusion of low glycemic index carbohydrates is a critical component of dietary management. Ongoing weight evaluation and self-monitoring of blood glucose (BG) levels are necessary to assess dietary effectiveness. Engaging in physical activity can further support BG control, with walking being the most practical and beneficial form of exercise for most women. Brown et al. [7].

In the context of specific conditions such as gestational diabetes, self-efficacy plays a vital role in guiding patients toward adherence to dietary recommendations, physical activity regimens, and glucose monitoring routines. Evidence from selected patient populations indicates that high self-efficacy is closely linked to more effective self-management. However, intervention studies that aim to enhance self-management behaviors by boosting self-efficacy have yielded mixed results, suggesting that improvements in self-efficacy alone may not be sufficient and might require integration with other behavioral support strategies Cheong et al. [8].

By using contemporary communication technology to close the distance between patients and providers, telehealth and telemedicine are completely changing the way healthcare services are provided. These methods make healthcare more affordable, accessible, and efficient by allowing care to be given without needing to be in close physical contact. Telemedicine, which includes clinical applications like virtual consultations and remote patient monitoring, as well as non-clinical elements of the healthcare system like administrative meetings, professional education, and training, are all included in the term “telehealth.” The prefix “tele” comes from the Greek word “at a distance,” which captures the spirit of providing care from a distance, Anawade et al. [9].

Nursing services have changed significantly within this context as well. The delivery, administration, and coordination of nursing care using telecommunications technologies is the special focus of tele-nursing, a subset of telehealth. It increases access to high-quality treatment by allowing nurses to communicate with patients across regional boundaries. Tele-nursing is frequently used to provide health education and counseling, do nursing teleconsultations, examine and interpret laboratory and diagnostic results, assist patients with self-care, and work with doctors to carry out treatment plans Bulto [10].

Beyond its clinical uses, tele-nursing also plays an important role in chronic disease management, post-operative follow-up, health promotion, and preventive care. It has the potential to enhance patient engagement, improve adherence to care plans, and reduce unnecessary hospital visits. By integrating tele-nursing into healthcare systems, nursing professionals can extend their reach, personalize care, and support patients in real time, ultimately improving health outcomes and patient satisfaction Ezeamii [11]. Telephone support, a practical tele monitoring method, offers health education and guidance to help patients manage their conditions through medication adherence, physical activity, and proper nutrition, thereby minimizing disease complications. It is a cost- and time-saving option, particularly for patients living far from healthcare facilities, and remains easily usable by all age groups. Research indicates high satisfaction among patients and notable efficiency gains for providers. In the context of gestational diabetes, nurses utilize telephone-based education and follow-up to encourage healthy lifestyle compliance, work alongside physicians in screening, diagnosis, and treatment, and integrate research findings into practice to enhance outcomes for both mothers and infants Ben-Assuli [12].

### Significance of the study

One of the main causes of maternal morbidity and mortality during the perinatal period is known to be gestational diabetes mellitus, or GDM. Many women experience both the joy of expecting a new life and the worry that their health and the health of their unborn child may be at jeopardy when they receive this diagnosis. Around 4.7% of pregnant women in Egypt have GDM, but the prevalence varies between 1% and 14% worldwide (WHO, 2011) (Upper Egypt Diabetes Association, UEDA, 2013), Wicklow & Retnakaran [13]. The primary line of action for managing GDM is lifestyle change, with an emphasis on weight control, appropriate physical activity, and balanced nutrition. Empowering women via educational initiatives that boost self-efficacy and empower them to actively manage their illness is equally vital. Maintaining appropriate blood glucose levels, reinforcing healthy behaviors, and reducing the risk of problems for both mother and newborn require ongoing assistance and follow-up. In the end, improving outcomes in pregnancies impacted by GDM requires early discovery, patient education, and ongoing engagement in care, Le et al. [14].

With an emphasis on enhancing the wellbeing of expectant mothers with GDM and guaranteeing safe, healthy pregnancy outcomes, an increasing number of healthcare organizations have been actively creating and putting into practice strategies to address the complex challenges of diabetes care in recent years O’Reilly et al. [15]. Also, the Upper Egypt Diabetes Association (UEDA) and reproductive health facilities at the obstetric departments of Upper Egypt Universities are important participants in this endeavor. They offer integrated services that combine clinical care, patient education, and continuing support O’Reilly et al. [15].

Many of the obstacles that restrict access to antenatal services, including lack of specialized care, time constraints, and geographic distance, can be creatively addressed by tele-nursing, one of the twelve fundamental telehealth functions recognized by the World Health Organization (2011) [16] and backed by strong evidence. Telephone-based health education, counseling, and follow-up are commonly utilized during pregnancy and the postpartum phase, and tele-nursing has been successfully employed to manage high-risk pregnancies on a global scale. Tele-nursing has not yet been included into Tanta City’s healthcare system, despite its demonstrated advantages outside, and there are currently insufficient randomized controlled trials evaluating its efficacy for women with GDM in this setting (Lyu et al. [17]).

Considering this disparity, the current study aims to compare the effects of tele-nursing and traditional nursing care as a novel strategy for improving the lifestyle, self-efficacy, and satisfaction of women with gestational diabetes. The trial intends to enhance glycemic control, encourage adherence to suggested lifestyle modifications, and ultimately lessen maternal and newborn problems linked to GDM by providing structured, nurse-led telephone support.

### Aim

To assess the effects of tele-nursing with standard nursing care as a novel strategy for improving the lifestyle, self-efficacy and satisfaction of women with gestational diabetes.

### Research hypothesis

Gestational diabetic women in tele-nursing group are expected to experience better knowledge, healthy lifestyle, self -efficacy and satisfaction than women in traditional group.

### Operational definitions

#### Tele-nursing

Tele-nursing uses telecommunications and technology to provide nursing care, education, and consultation remotely, allowing nurses to deliver services without the patient being physically present and overcoming geographic and logistical barriers.

#### Traditional nursing interventions

It involves direct, face-to-face care provided in healthcare settings or through in-person home visits, following the nursing process to address patients’ physical, psychological, and educational needs.

#### A healthy lifestyle

Consists of regular habits and choices that support physical, mental, and social well-being, reduce disease risk, and prevent premature death. It includes balanced nutrition, consistent physical activity, sufficient rest, stress management, and avoiding harmful behaviors like smoking, excessive drinking, and inactivity.

#### Maternal satisfaction

Refers to a mother’s perceived fulfillment, comfort, and positive experience with the healthcare services she receives during pregnancy, childbirth, and the postpartum period.

### Methods

#### Design

A study adopted a quasi-experimental research design which has been used, which is a quantitative method to assess an intervention’s effect when random group assignment isn’t feasible. It aims to determine cause-and-effect by comparing outcomes between tele-nursing and traditional care groups.

#### Setting

Three large hospitals representing various healthcare settings—Tanta University Hospital (Ministry of Higher Education), El-Menshawy General Hospital (Ministry of Health), and El-Mabra Hospital (Health Insurance Organization)—were used for the study’s antenatal outpatient care units.

Each setting included a single room equipped with two desks, one for nurses and the other for doctors alongside an examination table and an ultrasound machine.

#### Subjects

200 pregnant women with GDM were chosen as a purposive sample from the designated settings based on.

- Inclusion criteria: women who were literate, between 24- and 28-weeks’ gestation, had access to a telephone with internet connection, had no prior medical or obstetric complications, and consented to participate.
- Exclusion criteria included previous pregnancy loss, preterm delivery, and lack of telephone access.

The participants who agreed to participate were split evenly into two groups:

- **Tele-nursing group (*n* = 100)**: Received routine care plus a tele-nursing program involving telephone follow-up support.
- **Traditional care group (*n* = 100)**: Received routine nursing care provided at the clinic.

### Sample size

The sample size was determined using the Epi-info 7 software, based on a 95% confidence level, 80% research power, and a 5% margin of error. Consequently, 200 pregnant women with a diagnosis of gestational diabetes were determined to be the necessary sample size.


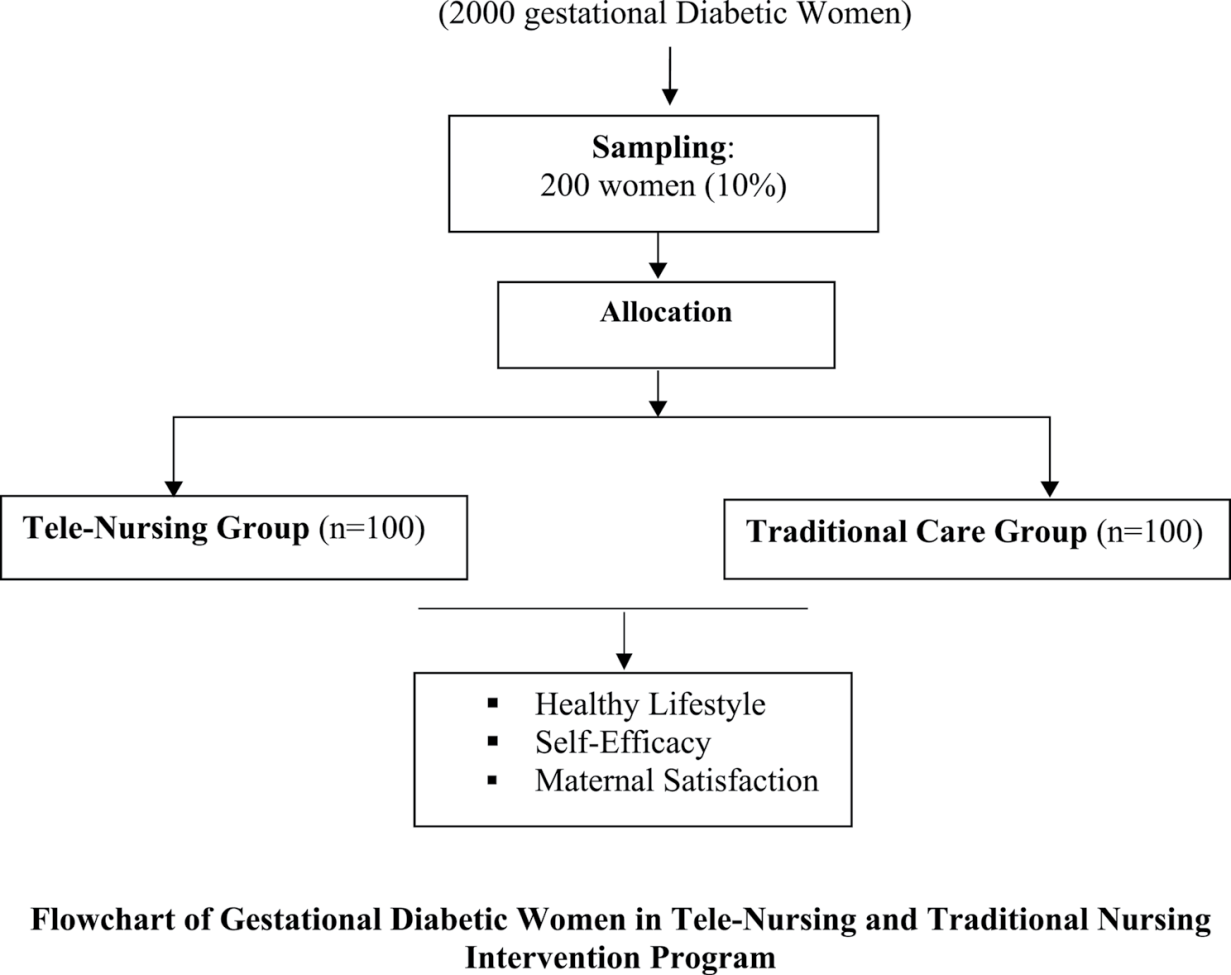


### Tools of data collection

Three tools were used to collect the necessary data for this study as follows: -

**Tool (I): **Structured interview questionnaire for knowledge assessment. Following a survey of recent related literature, researchers built it (O’Reilly et al. [15]). It was employed to evaluate the pregnant women’s understanding of GDM. There were three components to this tool.

**Part 1**: Sociodemographic Information: This section is used to gather information about the individual characteristics of the groups under study, including age, place of residence, educational attainment, occupation, and phone number.

**Part 2**: The pregnant women under study’s obstetric history, which included information on their gestational age, gravidity, parity, and manner of last delivery.

**Part 3**: Evaluation of the Researched Pregnant Women’s Knowledge of GDM: It was utilized to evaluate the pregnant women’s overall understanding of GDM, including its definition, symptoms, risk factors, and sequelae.

It also contained information on healthy lifestyle practices like eating the right foods, exercising, and being physically active, as well as treatment plans, prescription drugs, insulin injections, blood glucose monitoring, and the significance of postpartum follow-up. Every knowledge question had a score of one for a right response and zero for a wrong or “don’t know” response.

The aggregate of the scores, which ranged from 0 to 26, was then transformed into a percentage. Knowledge levels were categorized as low (less than 60%), moderate (between 60% and 80%), or high (more than 80%) based on this percentage.

**Tool II: Health Promotion Lifestyle Profile scale (HPLP)**: this tool has been adopted from Tanjani, P. et al. [18]; and Kuan et al. [19]. It included 52 items and six sub-scales: nutrition **(9 items)**, physical activity (**8 items**), health responsibility (**9 items)**, spiritual growth **(9 items)**, interpersonal support **(9 items)**, and stress management **(8 items)**. This questionnaire asks the respondents to indicate how often they adopt specific health-promoting behaviors on a 4-point Likert scale, with the options of “never” (1), “sometimes” (2), “routinely” (3) and always (4). In the present study, the subscales showed Cronbach’s alpha coefficients ranging from 0.70 to 0.88, with the overall scale demonstrating a reliability of 0.92. Walker and Hill-Polerecky [20] ) reported the original English version’s Cronbach’s alpha as 0.94 for the entire instrument and between 0.79 and 0.87 for its six subdomains. The tool was adapted and presented in the Arabic dialect to the participating mothers. Participants responded to the tool at baseline during the initial interview and again at 32 and 37 weeks of pregnancy. Higher scores over time reflect enhanced adoption of health-promoting behaviors.

The total scores range from 52 to 208, with higher total scores indicating a positive healthy lifestyle. (positive lifestyle ≥ 60% and negative lifestyle < 60%).

**Tool III: The Self-Efficacy of Health Behaviors (SEHB) scale**: It was adapted from Gandoy-Crego et al. [21]; and Clemente [22], , it included 25 items that measure the participant’s confidence in performing health-related behaviors across four domains: nutrition, exercise, psychological wellness, and health responsibility. Responses are given on a 4-point Likert scale from 1 (no confidence) to 4 (greater than 75% confidence). The total scores range from 25 to 100, with higher totals indicating stronger self-efficacy (satisfied self- efficacy ≥ 60% and unsatisfied self- efficacy < 60%). The mothers completed this scale at the initial interview (baseline) and subsequently at 32 and 37 weeks of gestation. The instrument demonstrated excellent reliability in this study, with a Cronbach’s alpha of 0.95 overall and subscale alphas ranging between 0.86 and 0.92.

**Tool IV: Women Satisfaction Likert scale regarding way of support for gestational diabetes mothers (Tele-nursing and traditional)**: It was adapted from Farrag & Metwely [23] and SH, et al. [24] to assess pregnant women’ satisfaction regarding Tele-nursing versus traditional program. The scale included 14 statements in the form of a three-point Likert scale as; The healthcare provider paid close attention to my concerns, The guidance given for managing gestational diabetes was clear and easy to follow, I was happy with how often I received support or communication, The healthcare provider treated me with respect and kindness, The support I received increased my confidence in managing my condition, The telephone follow-up in tele-nursing was convenient and easy to access, The in-person visits (for traditional care) met my needs effectively, I was satisfied with the timeliness of responses to my questions, The support I received addressed my emotional needs, The instructions given were practical and easy to follow, I felt involved in decisions about my care, he healthcare provider motivated me to maintain a healthy lifestyle, I felt reassured about my pregnancy after receiving support and Overall, I am satisfied with the care and support provided during my pregnancy.

A three-point Likert scale was used to grade each item, with 1 denoting “not satisfied,” 2 denoting “moderately satisfied,” and 3 denoting “satisfied.” The following was the range and grade for the overall satisfaction score. - Unsatisfied < 60%, - Moderate satisfied 60- < 75% - Content ≥ 75%.

## Method

### Administrative steps

- An official letter requesting permission to conduct the study was obtained from the Faculty of Nursing and submitted to the directors of the selected settings to secure their approval and cooperation.
- Content validity: All tools were tested for content validity by 5 experts in the field of Maternal and Neonatal Health Nursing.
- Data collection was conducted continuously from beginning of August 2025 to the end of October 2025, covering a period of three months to ensure adequate recruitment and thorough data gathering.
- Data collection was conducted during both morning and afternoon shifts and continued until the full target sample size was achieved.
- Data were initially collected from women in the traditional group to identify their needs, allowing the researchers to effectively prepare, plan, and implement tele-nursing intervention sessions centered on achieving healthy lifestyle, positive self-efficacy and maternal satisfaction.

### Operational procedures

The study was carried out through the following sequential steps:

### The actual study

The program was delivered by the researcher to ensure that both groups received comprehensive, consistent, and accurate information about GDM and its management through two approaches:

- The first approach was traditional, involving face-to-face meetings with women at the previously mentioned settings three days per week, scheduled according to times arranged by the facility managers.
- The second approach utilized technology, employing online tools such as Google Forms for data collection and health education sessions conducted via the Zoom application (telehealth and tele-nursing education).

### Development and implementation of the nursing intervention program

The process was structured into distinct phases to ensure systematic execution.

#### Assessment phase (Pretest)

During this initial phase, baseline data were gathered from all study participants prior to any intervention. The data collection was conducted using the specified questionnaires, which were administered individually to each participant to collect their socio-demographic data.

#### Planning and implementation phases

After assessing women’s needs during the initial phase, the researchers designed a comprehensive nursing intervention program tailored to improve knowledge, promote healthy lifestyle changes, and enhance control measures for GD management. The program specifically addressed gaps in understanding about GDM, covering topics such as its definition, risk factors, causes, clinical signs and symptoms, as well as strategies for prevention and effective management

- **For the tele-nursing group**, the educational content was delivered through four structured online sessions via the Zoom platform. Each session lasted between 30 and 45 min and was conducted as an interactive group discussion, allowing participants to engage actively, ask questions, and receive detailed explanations. Various teaching aids, including PowerPoint slides and educational videos, were incorporated to enrich the learning experience and facilitate comprehension. The researchers provided the tele-nursing group with telephone support, using a support booklet as a guide for each call. This booklet was created by researchers based on World Health Organization recommendations on gestational diabetes and supported by other scientific sources, with validation from experts. During each phone call, the mother was asked about any difficulties she was experiencing, and the researcher offered appropriate advice. The researcher also inquired whether the mother had implemented the guidance given previously. The discussions also covered essential health topics such as diet, physical activity, medication compliance, maintaining blood sugar within target levels, tracking and frequent self-monitoring of blood glucose, managing stress, and reminders about upcoming appointments. At the end of each call, mothers were encouraged to ask any questions they had. Calls were made once a week, averaging 4 calls per participant.
- **Also, for, the traditional face-to-face group** consisted of four sessions, each approximately 30 to 45 min long. Participants were provided with printed handouts written in straightforward language, accompanied by relevant images and illustrations to aid understanding. This format emphasized direct interaction and allowed for personalized guidance, reinforcing the educational content effectively.

#### Evaluation phase

Information related to the Healthy Promotion Lifestyle Profile (HPLP) scale, Self-Efficacy of Health Behaviors (SEHB) scale, antenatal visit frequency, and blood glucose levels was gathered from both the intervention and control groups at three distinct intervals: the initial baseline assessment, conducted during the first interview shortly after the diagnosis of gestational diabetes between 24 and 26 weeks of pregnancy and prior to any intervention; a follow-up at 32 weeks gestation; and a final evaluation at 37 weeks gestation. Furthermore, after delivery, both groups were evaluated to determine their satisfaction with the type of support they received for managing gestational diabetes throughout the antenatal period.

### Statistical analysis

The Statistical Package for Social Sciences (SPSS), version 18.0, was used for statistical analysis, while Epi-Info 6.04 was used for data entry. Both the coding and data entering stages involved the application of quality control procedures. The data was summarized using descriptive statistics, which displayed quantitative variables as means and standard deviations and qualitative factors as frequencies and percentages. Qualitative variables were compared using the Chi-square test. A p-value of less than 0.05 was deemed statistically significant, whereas p-values greater than 0.05 were deemed not significant. Values below 0.01 were deemed very significant.

## Results

Table 1 Illustrates the socio-demographic characteristics of the studied participants. It is showed that the tele-nursing group had a mean age of 19.30 ± 1.37 years, while the traditional group had a mean age of 18.99 ± 1.49 years.

Table 2 Demonstrates the HPLP scores which were measured at three points: baseline (24–28 weeks, first interview), 32 weeks, and 37 weeks of gestation. At the baseline assessment, before the intervention, there was no significant difference in the mean scores between the tele-nursing and traditional groups (*p* = 0.842). By the second interview at 32 weeks, a significant difference was detected (*p* = 0.004), and by the third interview at 37 weeks, the tele-nursing group demonstrated a highly significant improvement in mean HPLP scores compared to the traditional group (*p* = 0.000).

Table 3 Shows that before the intervention, the baseline measurements revealed no significant difference in average self-efficacy scores between both groups (*p* = 0.731). However, by the second interview (*p* = 0.003) and again in the third interview (*p* = 0.001), a statistically significant difference emerged, with the tele-nursing group achieving higher mean self-efficacy scores than the traditional group.

Table 4 Presents the distribution of the antenatal care visits from the start of the tele-nursing program, showing mean ± SD values of 12.00 ± 1.0 for the tele-nursing group and 8.2 ± 1.1 for the traditional group. This difference was statistically significant (*p* = 0.004), indicating that participants in the tele-nursing group attended more antenatal visits than those in the control group.

Table 5 Clarifies the fasting blood glucose measurements taken at baseline and at 32 weeks of gestation, with no statistically significant differences detected between the tele-nursing and traditional groups (*p* = 0.066 and *p* = 0.077, respectively). Over 37 weeks, however, a statistically significant difference was noted (*p* = 0.003) in favor of the tele-nursing group. For postprandial blood glucose levels, baseline assessment showed no significant difference (*p* = 0.120), while significant differences were recorded at 32 weeks (*p* = 0.005) and 37 weeks (*p* = 0.004) post-intervention, indicating better outcomes in the tele-nursing group.

**Fig. 1:**
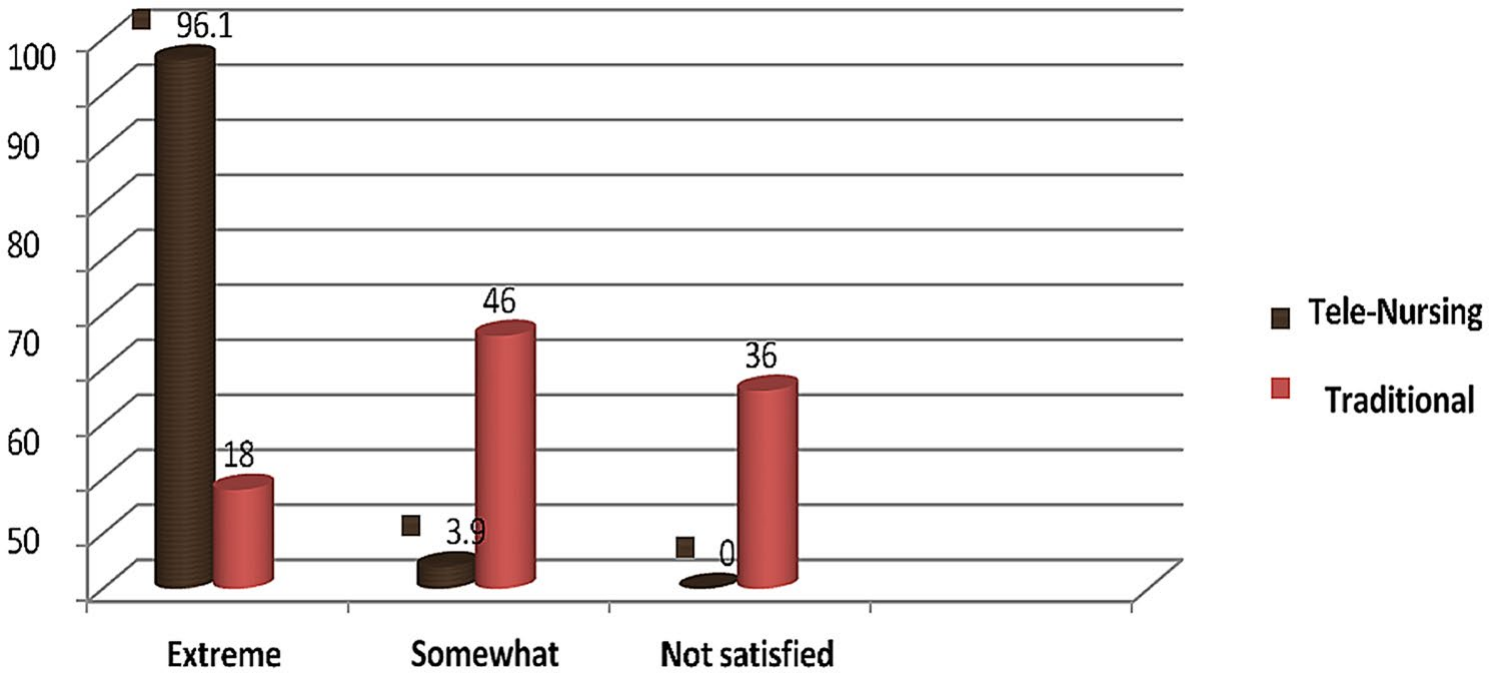
Distribution of gestational diabetic women’s according to satisfaction level in both groups

Figure 1 depicts the satisfaction of women with gestational diabetes concerning the type of antenatal support received (routine care alone versus routine care with telephone support). In the tele-nursing group, 96.1% reported being extremely satisfied, compared to just 18% in the traditional group. In contrast, dissatisfaction was reported by 36% of the traditional group, while no participants in the tele-nursing group expressed dissatisfaction. This difference in satisfaction between the two groups was statistically significant (*p* = 0.003).

**Table 1:** Percent distribution of both groups according to their socio demographic characteristics

Socio demographic characteristics	Tele-nursing group (*n* = 100)		Traditional group (*n* = 100)		Test of Sig.	P
	No.	%	No.	%		
**Age (years)** Range	20.0–45.0		19.0–46.0		U = 3420.5	0.129
Mean ± SD.	19.30 ± 1.37		18.99 ± 1.49			
**Place of residence**					18.182	0.164
Rural	30	30.0	32.0	32.0		
Urban	70	70.0	68.0	68.0		

**Table 2:** Total mean scores Of both groups according to their health promotion lifestyle profile scores during baseline, at second 32 wks

Assessment of Health Promotion Lifestyle Profile Mean Scores	Tele-nursing group (*n* = 100)	Traditional group (*n* = 100)	*P*-value
	Mean ± SD		
1st assessment (at 24–28 weeks gestation)	111.24 ± 10.02	109.23 ± 11.12	0.842
2nd assessment (at 32 weeks gestation)	136.14 ± 18.22	115.19 ± 09.36	0.004
3rd assessment (at 37 weeks gestation)	144.37 ± 21.25	118.22 ± 12.41	0.000

Of gestation and third (37 wks. Of gestation assessments

**Table 3:** Total mean scores of both groups according to their self-efficacy scores during initial, second and third assessments

Assessment of Self- Efficacy Scores	Tele-nursing group (*n* = 100)	Traditional group (*n* = 100)	*P*-value
	Mean ± SD		
1st assessment (at 24–28 weeks gestation)	59.03 ± 10.02	58.23 ± 11.12	0.731
2nd assessment (at 32 weeks gestation)	70.14 ± 12.22	61.19 ± 09.36	0.003
3rd assessment (at 37 weeks gestation)	73.37 ± 21.25	62.12 ± 12.41	0.001

**Table 4:** Total mean scores of antenatal care visits among the studied groups

Antenatal Care Visits of Gestational Diabetic Women	Tele-nursing group (*n* = 100)	Traditional group (*n* = 100)	*P*-value
Mean ± SD	12.00 ± 1.0	8.2 ± 1.1	0.004

**Table 5:** Total mean scores of both groups according to glucose levels during initial, second and third assessments

Assessment of Blood Glucose Level	Tele-nursing group (*n* = 100)	Traditional group (*n* = 100)	*P*-value
	Mean ± SD		
**1st assessment** **(at 24–28 weeks gestation)**			
Fasting	103.1 ± 15.02	101.01 ± 14.02	0.066
Postprandial	133.01 ± 12.01	138.06 ± 10.05	0.120
**2nd assessment (at 32 weeks gestation)**			
Fasting	100.19 ± 19.3	104 ± 15.8	0.076
Postprandial	123.18 ± 18.8	141.29 ± 18.7	0.005
**3rd assessment (at 37 weeks gestation)**			
Fasting	99.15 ± 17.02	105.15 ± 15.32	0.003
Postprandial	128.25 ± 15.01	132.58 ± 25.01	0.004

## Discussion

The risk of detrimental health outcomes for both the mother and her unborn child is significantly increased by GDM. These issues raise the expense of prenatal care, childbirth, and the length of time needed to recover after giving birth. Because of this, pregnancy is a crucial time to give women with GDM focused health education. A vital initial step in diabetes management is health education, which provides women with the knowledge, abilities, and useful strategies they need to manage the daily facets of self-care. This promotes improved health outcomes for the fetus and the mother Huter et al. [25]. The use of technology and communication platforms to provide care is known as tele-nursing. It is becoming a more contemporary method of improving nursing practice and is being used more in the treatment of different chronic illnesses, especially after COVID-19 struck and social distancing became necessary. To improve lifestyle, self-efficacy, and satisfaction among pregnant diabetes women, this study sought to compare the effects of tele-nursing with regular nursing care Jafarzadeh and associates [26].

In relation to the sociodemographic traits of the research participants. The average age of the tele-nursing group was 19.30 ± 1.37 years, whereas the average age of the traditional group was 18.99 ± 1.49 years. This was consistent with Eraky et al. [27], who reported that the participants’ performance improved statistically significantly following the implementation of the teaching program. Furthermore, this conclusion was corroborated by Xie, W., et al. [28], who noted that new technical assistance for the clinical management of GDM has been made possible by the advancement of information and communication technology.

The current study demonstrated no statistically significant difference between the two groups’ HPLP average scores during the baseline assessment, which was the initial interview conducted 24–28 weeks prior to the intervention. On the other hand, the study group’s scores at the second assessment (32 weeks) and the third exam (37 weeks) showed a notable improvement. The fact that both groups had access to the same information and support prior to the intervention helps to explain this improvement. However, the tele-nursing group’s participants benefited from further follow-up and individualized coaching through the tele-nursing program after the intervention. This ongoing remote support not only reinforced the importance of managing their condition but also increased their motivation to adopt and maintain a healthy lifestyle, thereby aiding in blood sugar control and safeguarding the wellbeing of both mother and her fetus.

The study of Maleki et al. [29], who carried out a randomized controlled experiment to assess the effect of mobile-assisted education on the blood sugar levels and health-promoting lifestyle of women with gestational diabetes, further supports this finding. According to their research, following 2, 4, and 6 months of regular telephone follow-up, the intervention group saw considerable improvements in quality of life, a decrease in GDM-related problems, and an increase in health-promoting lifestyle scores. These findings support the current study’s premise, which showed that women in the study group had considerably higher mean scores on the health promotion lifestyle profile than women in the control group.

In a recent study, Araby et al. [30] investigated how a self-care program based on the PRECEDE–PROCEED model affected the results for both the mother and the fetus in women with gestational diabetes. Comparing participants who received structured telephone support to those who did not, the results showed that the former were more likely to comply with healthy dietary patterns, exercise more frequently, practice effective prenatal self-care, and manage stress better. The authors stressed that the lifestyle intervention program included advice on nutrition, exercise, social cognitive approaches, and motivational interviewing techniques. It was administered via telephone follow-up by qualified coaches. The overall health-promoting lifestyle profile of women with gestational diabetes was improved and healthier lifestyle behaviors were fostered thanks to this multidimensional approach.

However, the current study’s findings contrast with those of Rady et al. [31], who looked at how diabetic patients’ lifestyle and clinical state were affected by tele-nursing (phone-based follow-up) in conjunction with an educational package. Participants in their study were divided into two groups: a control group and a telephone intervention group. One of the researchers administered the intervention, which included weekly calls that included continuing education, dietary compliance reinforcement, physical activity encouragement, medication regulation, and blood glucose monitoring. Their results showed no statistically significant change in the average Health-Promoting Lifestyle Profile (HPLP) ratings between the two groups, which contrasts with the findings of the current study.

Furthermore, the current study showed that significant and statistically significant improvements were seen in the study group at 32 and 37 weeks of gestation, even though there was no statistically significant difference in the mean scores on the self-efficacy scale between the two groups at baseline before the intervention. This improvement can be ascribed to the researchers’ continuous, structured tele-nursing support, which encouraged the mothers to maintain lifestyle modifications meant to protect their own and their fetus’ health while also reaffirming their confidence in their capacity to adhere to the recommended program. Furthermore, most of these women were from rural communities, where cultural values place a high emphasis on respect for and compliance with the guidance of trusted helpers. This cultural disposition may have played a pivotal role in their commitment to carefully following the advisors’ instructions.

The results of the current study are in line with those of Gebauer et al. [32], who looked at how academic self-efficacy varies among cultures and what factors influence it in various socialization contexts. Additionally, their findings showed that the groups’ average self-efficacy levels differed significantly. The current findings, however, contradict those of El-Ansary & Fouad [33], who investigated how instructional sessions affected pregnant women with gestational diabetes’s knowledge, attitudes, and self-care behaviors. The self-efficacy scores of the intervention and control groups in their study did not differ statistically significantly.

In terms of the distribution of antenatal care visits from the onset of the intervention, the results indicated that the mean ± standard deviation of visits among the tele-nursing group was significantly higher than that of the traditional group (*p* = 0.004). This notable difference may be explained by the persistent follow-up and reminders provided by the researchers, which ensured that women were aware of and prepared for each scheduled visit. Furthermore, the health education delivered emphasizing the critical role of antenatal visits, especially for women at higher risk helped strengthen their commitment to attending appointments. The ongoing communication and personalized support through tele-nursing further reinforced this awareness, motivating mothers to adhere closely to their antenatal care schedules.

This study’s findings are consistent with those of Olajubu et al. [34], who found that a straightforward phone call for a follow-up intervention increased adherence to attending prenatal treatment. Compared to the control group, the research group’s women were more than twice as likely to attend four or more prenatal care visits as advised.

There were no statistically significant differences between the two groups at the baseline assessment (*p* = 0.066) or at 32 weeks of gestation (*p* = 0.076), according to the study of fasting blood glucose levels. However, a statistically significant difference (*p* = 0.003) was found by 37 weeks, showing that the tele-nursing group had improved glucose management. Similarly, there was no significant difference in postprandial blood glucose levels at baseline (*p* = 0.120), but after 32 weeks (*p* = 0.005) and 37 weeks (*p* = 0.004), statistically significant differences appeared, favoring the tele-nursing group once more. These findings may be explained by the structured telephone follow-up sessions provided to the tele-nursing group, which reinforced compliance with dietary recommendations and physician-prescribed drug therapy, encouraged consistent participation in physical activity, enhanced overall awareness about the condition, and supported behavioral change by replacing harmful habits with healthier lifestyle choices.

The current findings are consistent with those of Elgaphar & El Gafar [35], who looked at how tele-nursing—phone-based follow-ups—affects diabetes patients’ glucose control, healthy lifestyle, and self-efficacy. According to their findings, there was a notable decrease in HbA1c levels and a notable increase in postprandial blood sugar control. Participants also showed positive changes in their eating habits, increased physical activity, improved medication adherence, adoption of healthier lifestyle choices, and heightened self-efficacy. These results provide more evidence of the beneficial effects of telephone-based follow-up in fostering patient glucose control and general health.

El-Ansary & Fouad’s [33] findings, on the other hand, show that telephone-based interventions did not significantly lower fasting glucose levels in the intervention group when compared to the control group. This contrasts with the results of the current study. The current study showed that maternal satisfaction ratings were significantly greater during the prenatal period for women with gestational diabetes who received additional telephone follow-up (*p* = 0.004). This could be explained by the sense of reassurance and emotional support mothers experienced, knowing there was a dedicated person consistently available to assist, guide, and encourage them without showing fatigue or disinterest. Their trust was probably reinforced, their participation in self-care activities increased, and their entire pregnancy experience was enhanced by the tailored follow-up. These results align with those of Farrag & Metwely [23], who found that telephone follow-up programs have a beneficial impact on women’s satisfaction with the supportive antenatal care delivery approach.

### Recommendations

- Present the study’s findings to decision-makers in governmental and non-governmental organizations so they may embrace the methodology and give it top priority as part of plans to improve the standard of care for expectant mothers, especially those who are at high risk. The integration of telephone follow-up services into maternal and child health (MCH) should get particular attention.
- Further research:
  - Investigate the impact of tele-nursing support on pregnancy outcomes.
  - Explore the factors influencing the implementation and utilization of tele-nursing services.

### Limitations of the study

1. The expenses incurred from conducting telephone calls to participants were considerable.
2. Several participants did not respond to initial calls, necessitating repeated attempts by the researchers. This process was time-intensive and challenging.

## Conclusion

The findings of the study supported the research hypothesis as it showed a statistically significant enhancement in health-promoting lifestyle profile average scores, confirming that telephone support through a tele-nursing program effectively improves self-efficacy, lowers blood glucose levels particularly postprandial and boosts adherence to scheduled antenatal visits among women in the tele-nursing group. Additionally, participants who received the tele-nursing intervention reported markedly higher levels of satisfaction compared with those in the traditional group. Based on these positive effects, the study concludes that effective control of gestational diabetes can be achieved not solely through individualized learning within clinical settings, especially in underserved areas where resources are limited and patient loads are high but also through the adoption of supportive strategies such as tele-nursing. This approach ensures continuous monitoring, tailored guidance, and sustained motivation, bridging gaps in care and addressing barriers posed by time constraints and overcrowded healthcare environments.

## Data Availability

The datasets used and/or analyzed during the current study are available from the corresponding author on reasonable request.

## References

[1] Benton M, Bird J, Pawlby S, Ismail K. The impact of gestational diabetes mellitus on perceived mother-infant bonding: a qualitative study. J Reprod Infant Psychol. 2025;43(2):487–500. 10.1080/02646838.2023.2239834. Epub 2023 Jul 26. PMID: 37493446; PMCID: PMC11854049. 37493446 10.1080/02646838.2023.2239834 PMC11854049

[2] Mittal R, Prasad K, Lemos JR, Arevalo G, Hirani K. Unveiling gestational diabetes: an overview of pathophysiology and management. Int J Mol Sci. 2025;26(5):2320. 40076938 10.3390/ijms26052320 PMC11900321

[3] Zhong J, Zhang H, Wu J, Zhang B, Lan L. Analysis of risk factors associated with gestational diabetes mellitus: a retrospective case-control study. Int J Gen Med. 2024:4229–38. 10.2147/IJGM.S473972 PMC11416790 39308966

[4] Karkia R, Giacchino T, Shah S, Gough A, Ramadan G, Akolekar R. Gestational diabetes mellitus: association with maternal and neonatal complications. Medicina. 2023;59(12):2096. 38138200 10.3390/medicina59122096 PMC10744613

[5] El Sayed NA, McCoy RG, Aleppo G, Balapattabi K, Beverly EA, Briggs Early K, et al. Management of diabetes in pregnancy: Standards of care in diabetes—2025. Diabetes Care. 2025;48. 10.2337/dc25-S015 PMC11635054 39651985

[6] Zakaria H, Abusanana S, Mussa BM, Al Dhaheri AS, Stojanovska L, Mohamad MN, et al. The role of lifestyle interventions in the prevention and treatment of gestational diabetes mellitus. Medicina (Kaunas). 2023;59(2):287. 10.3390/medicina59020287 PMC9966224 36837488

[7] Brown J, Alwan NA, West J, Brown S, McKinlay CJ, Farrar D, Crowther CA. Lifestyle interventions for the treatment of women with gestational diabetes. Cochrane Database Syst Rev. 2017;5(5):CD011970. 10.1002/14651858.CD011970.pub2. PMID: 28472859; PMCID: PMC6481373. 28472859 10.1002/14651858.CD011970.pub2 PMC6481373

[8] Cheong L, Law LSC, Tan LYL, Amal AAA, Khoo CM, Eng PC. Medical nutrition therapy for women with gestational diabetes: current practice and future perspectives. Nutrients. 2025;17(7):1210. 40218968 10.3390/nu17071210 PMC11990351

[9] Anawade PA, Sharma D, Gahane S, Anawade Sr PA, Sharma DS. A comprehensive review on exploring the impact of telemedicine on healthcare accessibility. Cureus. 2024;16(3). 10.7759/cureus.55996 PMC11009553 38618307

[10] Bulto LN. The role of nurse-led telehealth interventions in bridging healthcare gaps and expanding access. Nurs Open. 2024;11(1):e2092. 10.1002/nop2.2092 PMC10784421 38268279

[11] Ezeamii VC, Okobi OE, Wambai-Sani H, Perera GS, Zaynieva S, Okonkwo CC, et al. Revolutionizing healthcare: how telemedicine is improving patient outcomes and expanding access to care. Cureus. 2024;16(7). 10.7759/cureus.63881 PMC11298029 39099901

[12] Ben-Assuli O. Cost-effectiveness, use and implementation of telehealth solutions for CHF and COPD: a systematic review using the PRISMA method. Health Policy Technol. 2025;101023.

[13] Wicklow B, Retnakaran R. Gestational diabetes mellitus and its implications across the life span. Diabetes Metabolism J. 2023;47(3):333–44. 10.4093/dmj.2022.0348 PMC10244196 36750271

[14] Le DC, Vu TB, Tran TN, Nguyen TL, Nguyen TB, Nguyen DC, Hoang VT. The effectiveness of lifestyle changes in glycemic control among pregnant women with gestational diabetes mellitus. Medicina. 2023;59(9):1587. 37763706 10.3390/medicina59091587 PMC10537217

[15] O’Reilly SL, McAuliffe FM, Geraghty AA, Burden C, Davies A. Implementing weight management during and after pregnancy to reduce diabetes and CVD risk in maternal and child populations. Proc Nutr Soc. 2025;84(1):24–35. 38037711 10.1017/S0029665123004883

[16] World Health Organization. Introduction to tele-nursing, 2011.

[17] Lyu M, Zhao Q, Yang Y, Hao X, Qin Y, Li K. Benefits of and barriers to telehealth for the informal caregivers of elderly individuals in rural areas: A scoping review. Aust J Rural Health. 2022;30(4):442–57. 35460580 10.1111/ajr.12869

[18] Tanjani PT, Azadbakht M, Garmaroudi G, Sahaf R, Fekrizadeh Z. Validity and reliability of health promoting lifestyle profile II in the Iranian elderly. Int J Prev Med. 2016;7(1):74. 27280010 10.4103/2008-7802.182731 PMC4882969

[19] Kuan G, Kueh YC, Abdullah N, Tai EL. M. Psychometric properties of the health-promoting lifestyle profile II: cross-cultural validation of the Malay Language version. BMC Public Health. 2019;19(1):751. 31196195 10.1186/s12889-019-7109-2 PMC6567916

[20] Walker SN, Hill-Polerecky DM. Psychometric evaluation of the Health-Promoting lifestyle profile II. Omaha: Unpublished Manuscript, University of Nebraska Medical Centre; 1996.

[21] Gandoy-Crego M, Clemente M, Gómez-Cantorna C, González-Rodríguez R, Reig-Botella A. Self-efficacy and health: the SEH scale. Am J Health Behav. 2016;40(3):389–95. 27103418 10.5993/AJHB.40.3.11

[22] Clemente M, Gómez-Cantorna C. Self-efficacy and health: the SEH scale. Am J Health Behav. 2016;40:389–95. 27103418 10.5993/AJHB.40.3.11

[23] Farrag RE, Metwely S. Effect of tele-nursing services on healthy lifestyle and self-efficacy among gestational diabetes women. Int J Nov Res Healthc Nurs. 2016;3(1):129–40.

[24] SH H, AB H. Knowledge and satisfaction among mothers with gestational diabetes. Egypt J Health Care. 2018;9(4):1–9.

[25] Huter K, Krick T, Domhoff D, Seibert K, Wolf-Ostermann K, Rothgang H. Effectiveness of digital technologies to support nursing care: results of a scoping review. J Multidiscip Healthc. 2020;13:1905–26. PMID: 33328736; PMCID: PMC7734078. 33328736 10.2147/JMDH.S286193 PMC7734078

[26] Jafarzadeh F, Rahmani F, Azadmehr F, Falaki M, Nazari M. Different applications of telemedicine-assessing the challenges, barriers, and opportunities-a narrative review. J Family Med Prim Care. 2022;11(3):879–86. 35495787 10.4103/jfmpc.jfmpc_1638_21 PMC9051697

[27] Eraky Mahmoud L, Abdel Hakeem Hasneen S, El-Bana M. Abd El-wahab Afifi Araby, O. Effect of instructional package on knowledge and attitudes among gestational diabetic women. J Nurs Sci Benha Univ. 2024;5(2):74–92.

[28] Xie W, Dai P, Qin Y, Wu M, Yang B, Yu X. Effectiveness of telemedicine for pregnant women with gestational diabetes mellitus: an updated meta-analysis of 32 randomized controlled trials with trial sequential analysis. BMC Pregnancy Childbirth. 2020;20(1):198. 32252676 10.1186/s12884-020-02892-1 PMC7137255

[29] Maleki M, Mousavi P, Abedi P, Rokhafrooz D, Maraghi E. Effect of mobile-assisted education on health promoting lifestyle and blood sugar of women with gestational diabetes: a randomised controlled trial. BMJ Nutr Prev Health. 2023;6(2):310. 38618532 10.1136/bmjnph-2023-000802 PMC11009530

[30] Araby A, Abdel-Wahab O, Ali HA, Abd Elmordy ZR. Effect of self-care program guided by PRECEDE–PROCEED model on maternal and fetal outcomes among gestational diabetic women. Tanta Sci Nurs J. 2024;34(3).

[31] Rady Magbool F, Eltoony F, Abd El-Nasser Ali L, G., Hassan Hussein A. Effect of tele-nursing (phone-based follow up) and educational package on lifestyle and clinical status for diabetic patients. Egypt J Health Care. 2021;12(1):881–91.

[32] Gebauer MM, McElvany N, Köller O, Schöber C. Cross-cultural differences in academic self-efficacy and its sources across socialization contexts. Soc Psychol Educ. 2021;24(6):1407–32.

[33] El-Ansary ES, Fouad S. Effect of educational sessions on knowledge, attitude and self-care practices among pregnant women with gestational diabetes. Egypt J Health Care. 2020;11(3):275–91.

[34] Olajubu AO, Fajemilehin BR, Olajubu TO, Afolabi BS. Effectiveness of a mobile health intervention on uptake of recommended postnatal care services in Nigeria. PLoS ONE. 2020;15:e0238911. 10.1371/journal.pone.0238911 PMC7489550 32925971

[35] Elgaphar S, El Gafar S. Effect of tele-nursing (phone-based follow-ups) on self-efficacy, healthy lifestyle, and glycemic control in diabetic patients. IOSR J Nurs Health Sci. 2017;6(3):67–7.

